# Investigations of the presence of caffeine in the Rudawa River, Kraków, Poland

**DOI:** 10.1007/s10661-015-4760-7

**Published:** 2015-08-12

**Authors:** Agnieszka Jagoda, Witold Żukowski, Barbara Dąbrowska

**Affiliations:** Faculty of Chemical Engineering and Technology, Cracow University of Technology, ul. Warszawska 24, 31-155 Kraków, Poland; Faculty of Environmental Engineering, Cracow University of Technology, ul. Warszawska 24, 31-155 Kraków, Poland

**Keywords:** Anthropogenic contaminants, Caffeine, Surface water monitoring

## Abstract

Caffeine concentration in surface water (Rudawa River, Kraków, Poland) has been being investigated since 2011. The method applied for investigations was developed in 2011, and the first series of measurements of caffeine concentration in surface water began in 2011. Caffeine concentration was determined by the gas chromatography-mass spectrometry (GC-MS) method. Solid phase extraction (SPE) was used to enrich the concentration of caffeine in water samples. As an internal standard, the caffeine isotope ^13^C_3_ in methanol (Sigma Aldrich) was used. The values of four additional parameters (concentration of nitrates, biochemical oxygen demand after 7 days, number of *Escherichia coli* and number of *Enterococcus faecalis*) were determined for the water sample analyzed. Caffeine was detected in all studied samples. The control series of measurements during 2011–2014 confirmed that caffeine is present in Rudawa River water and that the concentration of this substance in Rudawa River ranges from 14.0 to 852.0 ng/dm^3^. There is no correlation between the concentration of caffeine and the concentration of other anthropogenic contaminants determined in water.

## Introduction

Available information on the presence of pharmaceutical and personal care products (PPCP) in surface water is still very limited but the occurrence of these substances in the natural aquatic environment is recognized as an emerging issue due to the potential adverse effects these compounds pose to aquatic life and humans (Franke et al. 2005; Caliman and Gavrilescu 2009).

Wastewaters generated by households are sources of anthropogenic pollution of surface water. Domestic wastewater is formed in areas of human habitation and contains fecal matter which is produced by human organisms. Its composition is also connected with everyday personal hygiene and the functioning of households, such as preparing meals, washing dishes, laundering, and cleaning. Surface water contamination by domestic wastewater is caused by a lack of treatment plants or the ineffective action of such plants (in older types, there is only a mechanical purification step without a chemical-biological step). Wastewater is also discharged to the environment by leaking septic tanks or is treated as manure and poured directly onto fields, from where it is washed by rain into surface waters. Domestic sewage enters the water supply and contaminates it with a variety of chemical compounds and microorganisms, including bacteria, viruses, funguses, and protozoa. In this way, many harmful substances and pathogenic microorganisms enter the water supplies. Additionally, various reactions which can cause a reduction in oxygen levels and changes in the pH and temperature of the watercourse environment may occur between the sewage components. This can destroy habitats for many organisms, plants and animals may die, and the water may become unsuitable for human consumption.

Caffeine (1,3,7-trimethyl-1H-purine-2,6(3H,7H)-dione, 3,7-dihydro-1,3,7-trimethyl-1H-purine-2,6-dione, 1,3,7-trimethyloxanthine, 1-methyltheobromine, theine, guaranine, mateine) is strongly associated with domestic wastewater (http://chemsub.online.fr/name/Caffeine.html). Caffeine is the world’s most widely consumed *psychoactive* substance. It stimulates the body and reduces fatigue, tiredness, and sleepiness. It is a component of medical preparations, coffee, tea, caffeinated sodas, and cosmetics. It is well metabolized; according to various sources up to 20 % of caffeine is removed from the body unchanged (Mandel 2002; Thorn et al. 2012). Caffeine is a potential indicator of domestic wastewater because it is clearly of anthropogenic origin and is often detected in wastewater and surface water. The major source of caffeine in domestic wastewater comes from unconsumed coffee, tea, and other drinks containing coffee, from washing machines, dirty dishes, or coffee-making facilities, and from expired, unwanted, or unused medicines which are thrown away (Seiler et al. 1999).

Caffeine is detected in surface and ground waters all over the world (Jagoda et al. 2010). Measurements made in ten European countries showed that it occurs in the Danube River and its tributaries. Fifty-two water samples were collected from the Danube and 50 samples from its tributaries. In each sample, caffeine was detected. Its average concentration in the Danube River waters was 137 ng/dm^3^ and in the tributaries 406 ng/dm^3^. The maximum amount of caffeine detected in the Danube River was 1467 ng/dm^3^ and in its tributaries 6798 ng/dm^3^ (Loos et al. 2010). Caffeine was also found in the Lippe River (Germany), which is a tributary of the Rhine River. Water samples were collected four times from 19 measuring points which were located along the watercourse. Caffeine concentration ranged from less than 10 to 420 ng/dm^3^ (Dsikowitzky et al. 2004a, b). Analysis of water from the Seine (France) carried out four times showed the presence of caffeine. The content of caffeine at the measuring points located at 200, 313, and 355 km downstream from Paris ranged from 3.2 to 186.9 ng/dm^3^ (Togola and Budzinski 2007). The hypothesis that caffeine can be used as an indicator of anthropogenic pollution seems to be confirmed by the results of analysis of water from the Upper Iguassu in the metropolitan region of Curitiba, Brazil. The caffeine concentration was found in a range from 1200 to 124,350 ng/dm^3^. Additionally, positive correlations between the concentrations of caffeine and two traditional monitoring parameters biochemical oxygen demand and fecal coliforms were found (Froehner et al. 2011).

The main aim of this study was to detect the presence of caffeine in the Rudawa River in Kraków, Poland, and to determine its concentration. Additionally, analysis of selected water quality parameters was made. Four indicators of water pollution were chosen to show the current pollution of watercourses: two physicochemical water parameters (the concentration of nitrate (nitrate (V)) and biochemical oxygen demand (BOD)) and two microbiological parameters (number of *Escherichia coli* and number of *Enterococcus faecalis*).

Caffeine concentration in surface water (Rudawa River, Kraków, Poland) has been being investigated from 2011. The method applied for investigations was developed in 2011, and the first series of measurements of caffeine concentration in surface water began in the same year. This series of measurements was chosen as an example of the application of the newly developed method of determinations of caffeine concentrations in surface water. Since that time, the concentration of caffeine in the Rudawa River has remained in the same range, varying from 14.0 to 852.0 ng/dm^3^.

In Poland, research on and evaluation of the quality of surface waters is carried out by the State Environment Monitoring (SEM-Państwowy Monitoring Środowiska PMŚ) as dictated by Article 155a Paragraph 2 of the Act of 18 July 2001—Water Law (Dz. U. 2014r. poz. 659). Research on surface water quality concerning physico-chemical elements, chemical, and biological elements is the responsibility of the provincial environmental protection inspector. In these regulations, there is no provision for the determination of the concentration of caffeine in surface water by water quality monitoring stations.

## Study area

The Rudawa River is a left-bank tributary of the Vistula River flowing in southern Poland, with its mouth located in the urban setting of Kraków, at kilometer 75.4 along the Vistula course. The Rudawa catchment area is approximately 318 km^2^ and is situated in six communities; one city (Kraków), two urban-rural areas (Krzeszowice, Trzebinia), and three rural areas (Jerzmanowice-Przeginia, Wielka Wieś, Zabierzów). There are protected areas in the Rudawa catchment area, including the Kraków Valleys Landscape Park, the Tenczynek Landscape Park, and places included in the Natura 2000 network (Jurassic Valleys) (www.natura2000.gdos.gov.pl/natura2000/; www.zpkwm.pl/zespol-parkow/o-nas.html).

**Fig. 1:**
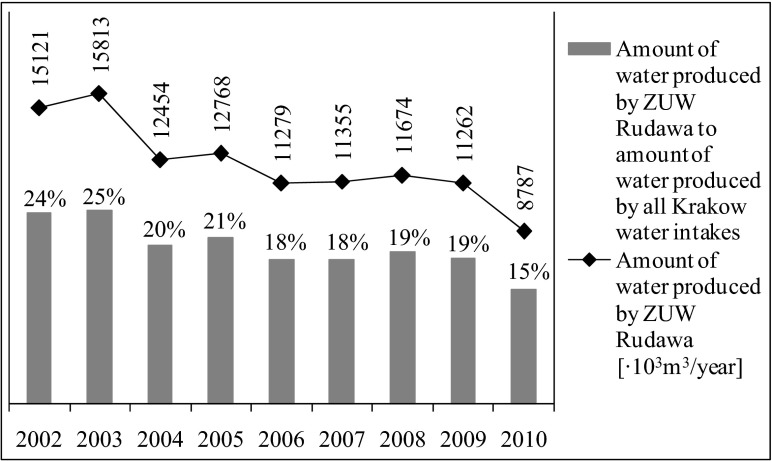
Characterization of the “Rudawa” Water Treatment Plant (www.bip.krakow.pl/?id=509)

On the Rudawa River, about 9 km from its mouth at the Vistula River, water intake for the Water Treatment Plant “Rudawa” (ZUW “Rudawa”) is located. It supplies water to the north-western part of Kraków and is the second largest producer in this area. In the years 1983–1985, it produced over 100,000 m^3^ of water per day. Currently, the plant capacity is about 55,400 m^3^ per day and represents 15 % of the total amount of water produced by all Kraków city intakes. The amount of water produced by the ZUW “Rudawa” and its contribution to the total quantity of water supplied to the waterworks in Krakow in recent years is presented in Fig. 1 (www.bip.krakow.pl/?id=509).

The Voivodeship Inspectorate for Environmental Protection (VIEP) in Kraków (WIOŚ w Krakowie) is a part of the State Monitoring of the Environment, which is the system of receiving, gathering, transforming, and making available information on the environment. VIEP monitors the Rudawa River. The VIEP measuring point is located at kilometer 9.3 of the river. Water samples taken at that location are representative for water in the Rudawa River along its course from Racławka to the Rudawa mouth (www.krakow.pios.gov.pl/inform.php).

The Ordinance of the Minister of Environment of Poland of 27 November 2002 on the requirements of surface waters used for supplying the population with drinking water (Journal of Laws of the Republic of Poland No. 204, position 1728, 2002) defines three classes of surface water quality and processes which should be carried out to condition water. Class A1 waters require a simple physical treatment, especially filtration and disinfection. Class A2 waters need a typical physical and chemical treatment, in particular pre-oxidation, coagulation, flocculation, decantation, filtration, and disinfection (final chlorination). Class A3 waters request very effective physical and chemical treatment, in particular, oxidation, coagulation, flocculation, decantation, filtration, adsorption on activated carbon, and disinfection (ozone, final chlorination) (Ordinance of the Minister of Environment of Poland of 27.11.^2002^). All these treatments are expensive, but this activity is necessary if surface water is to meet quality standards for human consumption. Differentiation of watercourses into categories depends on the results of water quality indicators obtained during analysis. Forty-four physiochemical and microbiological parameters describing the physicochemical and microbiological state of the water are determined along with reference methods for their measurements. It has been shown that in recent years, water from the Rudawa is class A3 or does not meet the requirements of any class (Table 1; www.krakow.pios.gov.pl/inform.php).

**Table 1:** Classification of the Rudawa River water in years 2007–2010 (Grenda and Bochnia 2011; www.krakow.pios.gov.pl/inform.php)

Year	General class of water quality	Class according to indicators	Water quality indicators [unit]	Annual values of indicator
2007	Does not meet A1, A2, A3	Does not meet A1, A2, A3	Phosphates [mg/dm^3^]	0.219–1.255 0.534
		A3	Total number of fecal coliforms [unit/100cm^3^]	2300–15,000 5475
			Total number of coliforms [unit/100 cm^3^]	2300–20,000 7725
2008	A3	A3	Phosphates [mg/dm^3^]	0.225–0.688 0.395
			Anionic surfactants [mg/dm^3^]	0.03–0.12 0.06
			Total number of fecal coliforms [unit/100 cm^3^]	900–9300 4900
			Total number of coliforms [unit/100 cm^3^]	2300–43,000 13,100
2009	Does not meet A1, A2, A3	A3	Suspension [mg/dm^3^]	5–35 16
		Does not meet A1, A2, A3	Total number of fecal coliforms [unit/100 cm^3^]	600–24,000 7213
			Total number of coliforms [unit/100 cm^3^]	3400–242,000 47,033
2010	A3	A3	Total number of coliforms [unit/100 cm^3^]	10,462–112,000 43,666
			Total number of fecal coliforms [unit/100 cm^3^]	1670–28,000 13,765

**Fig. 2:**
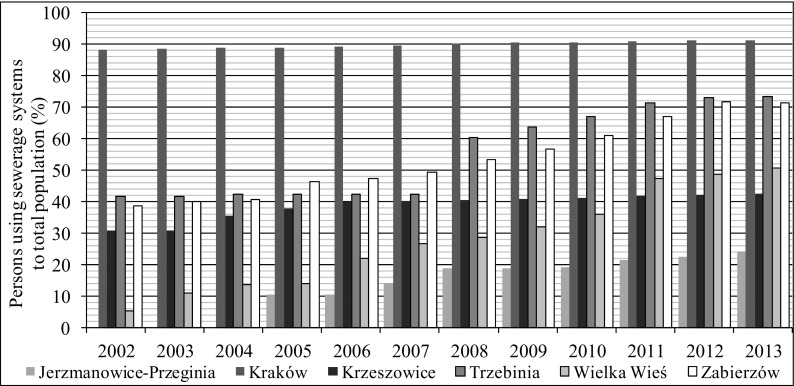
Persons using sewerage system to total population in selected municipalities in 2002–2013 (http://stat.gov.pl/bdlen/app/strona.html?p_name=indeks)

**Fig. 3:**
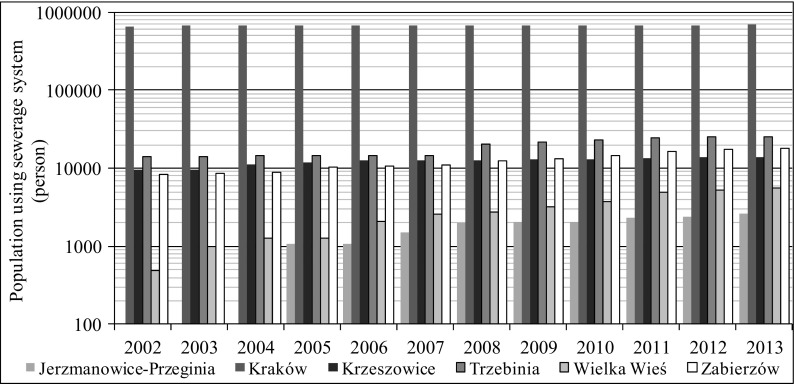
Population using sewerage system in selected municipalities in 2002–2013 (http://stat.gov.pl/bdlen/app/strona.html?p_name=indeks)

**Fig. 4:**
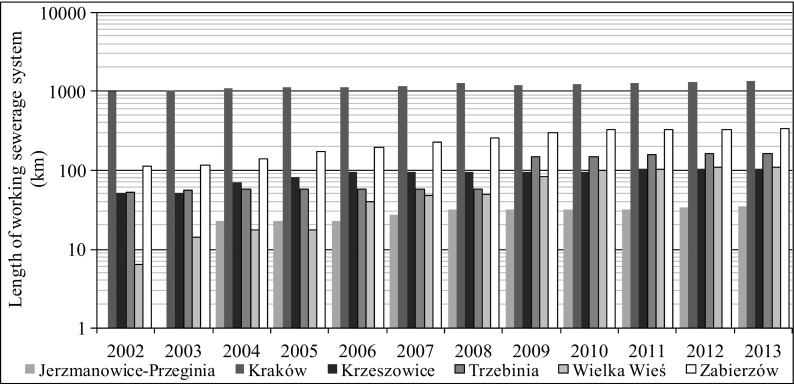
Length of working sewerage system in selected municipalities in 2002–2013 (http://stat.gov.pl/bdlen/app/strona.html?p_name=indeks)

**Fig. 5:**
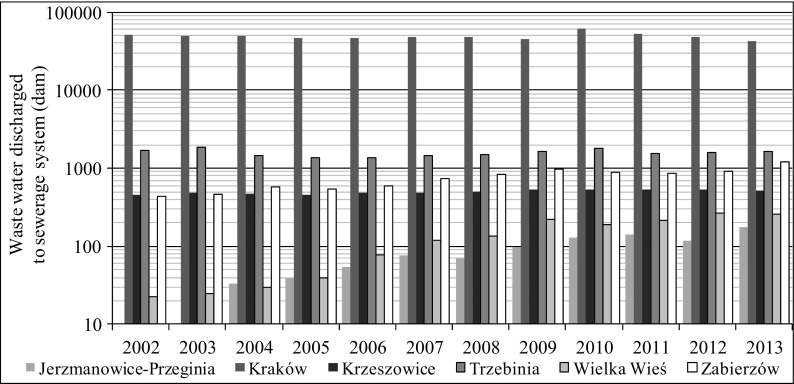
Waste water discharged to sewerage system in selected municipalities in 2002–2013 (http://stat.gov.pl/bdlen/app/strona.html?p_name=indeks)

A large disparity in the development of sewerage systems of particular Rudawa basin municipalities can be observed. Changes that took place in 2002–2010 regarding sewers and their usage are shown in Figs. 2, 3, 4, and 5 (http://stat.gov.pl/bdlen/app/strona.html?p_name=indeks).

In the Trzebinia municipality, the Rudawa River basin takes only a small fragment at its eastern part. Near the village Psary are numerous springs giving the beginning of Dulówka stream, which after passing 5.9 km flows to the Krzeszowice municipality. The Trzebinia municipality has an advanced sewer system. In 2010, 67 % of the population used the installation. Unfortunately, there are large disparities between urban and rural areas, with 96.5 % of the urban population using the sewer system, while only 24.7 % of the rural population do so—according to the Central Statistical Office in Poland (GUS Główny Urząd Statystyczny—CSO data). Wastewaters generated within the municipality of Trzebinia are treated by the Trzebinia-Siersza Sewage Treatment Plants and are sent to the Group of Wastewater Treatment Plants in Chrzanów. The Trzebinia-Siersza Sewage Treatment is a wastewater treatment plant with increased bio gene removal with a capacity of 3500 m^3^/day. The receiver of treated wastewater is the Kozi Bród stream, in the Biała Przemsza catchment (http://stat.gov.pl/bdlen/app/strona.html?p_name=indeks; www.wrotamalopolski.pl/root_BIP/BIP_w_Malopolsce/gminy/root_Krzeszowice/podmiotowe/Rada/Uchwaly/).

The Krzeszówka river flows through the Krzeszowice municipality. It comes near the village of Czerna at the confluence of two streams, the Czernka and Eliaszówka. Next, the Krzeszówka flows through Krzeszowice, where it joins the Miękinia and Dulówka streams, which flow from Trzebinia. Two tributaries, to the right the Olszówka stream and to the left the Filipówka stream, fall into the Dulówka just before its confluence with the Krzeszówka. The Krzeszowice municipality is poorly sewered. In 2010, only 40.8 % of the population used the installation, 25.3 % from rural and 74.9 % from urban areas. The Krzeszowice municipality has three wastewater treatment plants in Krzeszowice, Zalas, and Rudno. They use mechanical-biological purification methods. The plant capacity is 7000 m^3^/day at Krzeszowice, 125 m^3^/day at Rudno, and 400 m^3^/day at Zalas. Wastewater from Krzeszowice flows into the Krzeszówka River, while that from Rudno and Zalas to watercourses which are not from the Rudawa basin (from Rudno through a stream without a name into the Chechło River and from Zalas to the Rudno stream). Krzeszowice community inhabitants use also the Jerzmanowice-Przeginia community sewage treatment plant in Żary (www.jerzmanowice-przeginia.pl/prawo/gpgo.pdf).

The Jerzmanowice-Przeginia municipality includes starting points of three streams belonging to the Rudawa catchment, Będkówka, Racławka, and Szklarka. The municipality is poorly canalized; in 2010, only 19.2 % of the population used the sewer. The municipality has one biological sewage treatment plant located in Żary with a capacity of 350 m^3^/day. Treated effluents are discharged to the Racławka stream (www.wrotamalopolski.pl/root_BIP/BIP_w_Malopolsce/gminy/root_Zabierzow/podmiotowe/Rada/Uchwaly/).

The Rudawa River is formed in the Zabierzów community by the merger of the Krzeszówka River with the Racławka stream near of the village of Rudawa. Then, Rudawa is joined by left-hand tributaries, the Będkówka, Kobylanka (with Bolechówka), Kluczwoda, and Wędonka. The Zabierzów municipality is one of the best sewered. According to CSO in 2010, 61 % of the population used the sewer. The municipality has four municipal mechanical-biological sewage treatment plants in the villages of Balice, Niegoszowice, Radwanowice, and Zelków with capacities of 800, 800, 222, and 150 m^3^/day, respectively. Treated effluents are discharged from the Balice sewages to the Rudawa river (below the water intake for the Krakow city), from Niegoszowice to the Rudawa river, from Radwanowice through an unnamed stream to the Szklarka (Rudawka), and from Zelków to the Kluczwoda stream (www.wrotamalopolski.pl/root_BIP/BIP_w_Malopolsce/gminy/root_Zabierzow/podmiotowe/Rada/Uchwaly/; www.wrotamalopolski.pl/root_BIP/BIP_w_Malopolsce/gminy/root_Wielka+Wies/podmiotowe/Rada/Uchwaly/).

The Wielka Wieś municipality sewage system is very poorly developed. In 2010, only 36 % of the population used the sewer. Only five villages are connected to the sewer network (Giebułtów, Modlnica, Modlniczka, Szyce, and Wielka Wieś). Municipal sewage from Giebułtów, Szyce, and Wileka Wieś goes to a mechanical-biological treatment plant in Giebułtów with a capacity of 300 m^3^/day. Treated effluent is discharged to the Sudoł stream (Prądnik basin). Wastewaters from Modlniczka are discharged to the Krakow’s Sewage Treatment Plant. This sewage system does not belong to the part of the municipality where the Rudawa catchment is located (the Będkówka, Kluczwoda and Wędonka streams) (www.wrotamalopolski.pl/root_BIP/BIP_w_Malopolsce/gminy/root_Wielka+Wies/podmiotowe/Rada/Uchwaly/; http://www.pogodynka.pl/polska/podest/zlewnia_gornej_wisly).

The municipality of Kraków has a well-developed network of sewers which is used by 90.8 % of the population. The sewer system is composed of two separate systems (Płaszów and Kujawy) which have their own sewage treatment plants. Additionally, five local sewage treatment plants are located in Bielany, Kostrzewa, Sidzina, Skotniki, and Wadów. The Płaszów wastewater treatment plant has a capacity of 328,000 m^3^/day and Kujawy 8000 m^3^/day. The transport capacity values of local treatment plants are 225 m^3^/day for Bielany, 350 m^3^/day for Kostrzewa, 240 m^3^/day for Sidzina, 884 m^3^/day for Skotniki, and 563 m^3^/day for Wadów. All Kraków sewage treatment plant wastewaters are treated in a mechanical-biological way. The treated effluents are discharged to watercourses disgorging into the Vistula River. None of the watercourses belongs to the catchment of the Rudawa River (www.bip.krakow.pl/?id=509).

## Experimental

**Fig. 6:**
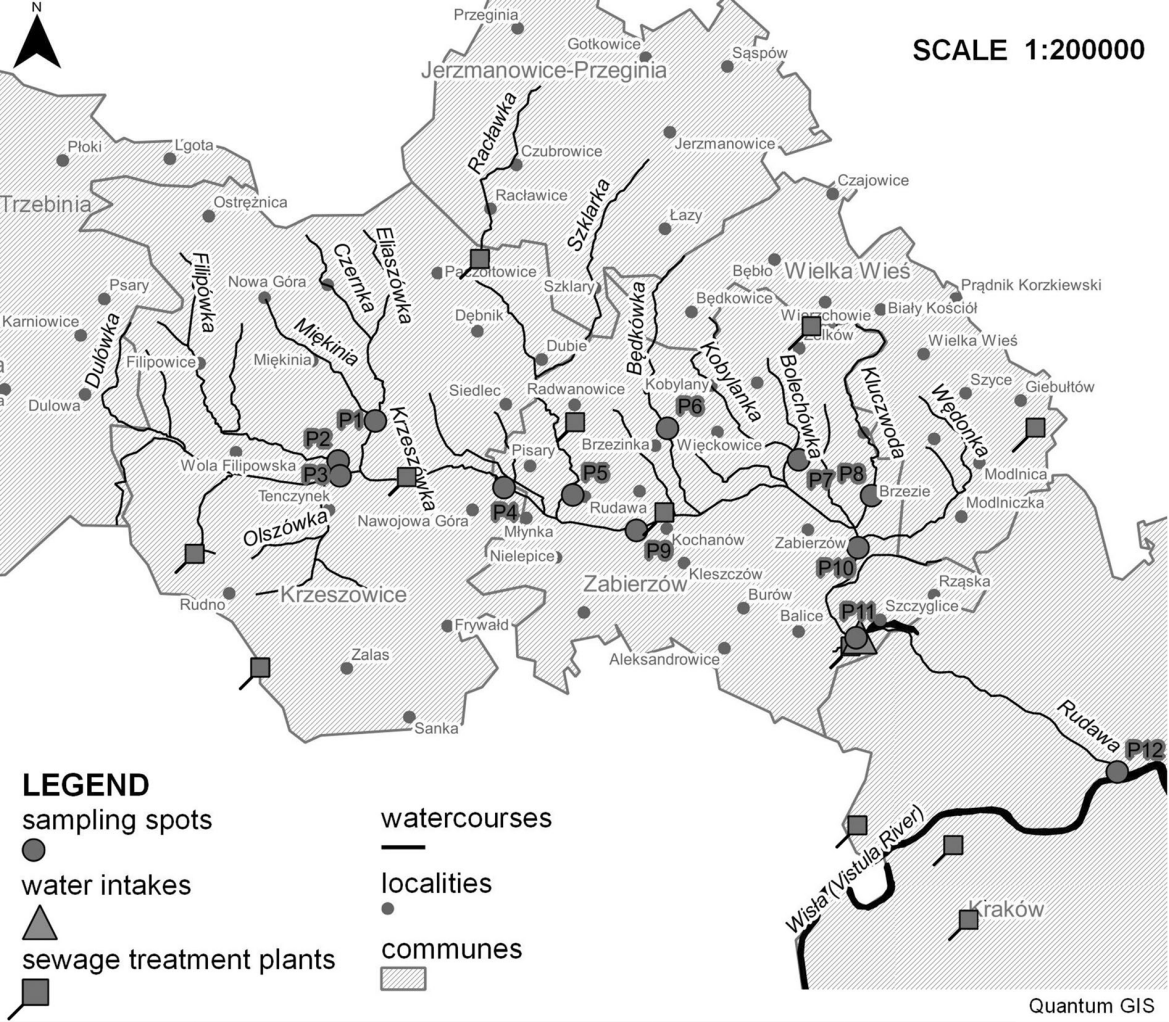
Localizations of the sampling points (www.gisplay.pl)

Water from the Rudawa River basin was analyzed. Water samples from 12 different locations were taken. The coordinates and description of the sampling points are collected in Table 2. The localizations of those points are shown in Fig. 6.

**Table 2:** Location of water sampling points

Creek	Sampling spot	Geographic coordinates	
		Latitude	Longitude
Krzeszówka	P1	50° 8′ 28.63′′ N	19°37′ 59.67′′ E
Filipówka	P2	50° 7′ 53.30′′ N	19°37′ 7.65′′ E
Dulówka	P3	50° 7′ 40.05′′ N	19°37′ 10.20′′ E
Krzeszówka	P4	50° 7′ 28.47′′ N	19°40′ 56.17′′ E
Rudawka	P5	50° 7′ 21.39′′ N	19°42′ 30.91′′ E
Będkówka	P6	50° 8′ 19.74′′ N	19°44′ 42.11′′ E
Kobylanka	P7	50° 7′ 50.57′′ N	19°47′ 42.57′′ E
Kluczwoda	P8	50° 7′ 17.63′′ N	19°49′ 22.85′′ E
Rudawa	P9	50° 6′ 49.54′′ N	19°43′ 57.80′′ E
Rudawa	P10	50° 6′ 31.93′′ N	19°49′ 3.16′′ E
Rudawa	P11	50° 5′ 11.86′′ N	19°48′ 59.44′′ E
Rudawa	P12	50° 3′ 10.25′′ N	19°54′ 56.55′′ E

### Sample collection

Samples were collected twice during 2011, on June 26 and on October 2. On both days when samples were collected, the temperature was below 20 °C. There was no rain, but the days were cloudy, and the sun started shining only in the afternoon. Samplings started in the afternoon at the spot which was located furthest away from Krakow (P1). The next measuring points were along the river paths to the city. Operations were ended just before the Rudawa River mouth (P12) in the early evening hours. Before the second samplings, there had been no rain for a long time and it had led to low water levels in the Rudawa River. Water from the river was taken from bridges by using a bucket hanging on a rope according to the standard procedures for water sampling methods and analysis. Water for caffeine analysis was stored in a 2.7-dm^3^ amber glass bottle.

### Physicochemical and microbiological analyses

Water samples which were intended for physicochemical and microbiological analyses were stored in a refrigerator until their delivery to the Sanitary and Epidemiological Station. Analyses were made according to the following methods: PN-EN ISO 10304-1:2009 (Nitrate), PN-EN 1899-2:2002 (BOD7), PB-LHK-12 (amount of *Escherichia coli* in 100 cm^3^ water), and PN-ISO 7899-2:2004 (amount of *Enterococcus faecalis* in 100 cm^3^ water). These indications of water quality parameters were made by the Sanitary and Epidemiological Station in Krakow.

### Caffeine analyses

An internal standard (caffeine isotope ^13^C_3_ in methanol, Sigma Aldrich) was added to each sample immediately after sampling. Samples were stored in a refrigerator according to the standard procedures for water sampling methods and analysis. Before analysis, each sample was warmed to room temperature. pH was elevated to 9 by adding a concentrated solution of sodium hydroxide (Polskie Odczynniki Chemiczne (POCH), Polish Chemical Reagents). Each sample was divided into three parts which were left undisturbed until the suspended solids in the water settled under the influence of gravity. Then, solid phase extraction was carried out using a VISIPRED 24TM DL (Supelco) set with 6 cm^3^ cartridges containing 500 mg of C18 sorbent (J.T. Baker). As conditioning solvents, the following solutions were used: methanol, water at pH 9 1:1 (*v*/*v*); ethyl acetate, acetone 1:1 (*v*/*v*); and again methanol, water at pH 9 1:1 (*v*/*v*) (each solvent from POCH, distilled water was elevated to pH 9 by adding a concentrated solution of sodium hydroxide (POCH)). Water samples were passed to the extraction columns with a mean flow rate ranging from 0.08 to 0.18 cm^3^/s. Then, the extraction columns were dried in a centrifuge with a speed of 4000 rpm for 20 min (centrifuge MPW-223e, MPW MED. INSTRUMENTS). One void volume mixture of solvents ethyl acetate: acetone 1:1 (*v*/*v*) (POCH), was used as an eluent. Solutions obtained in this way were concentrated in a vacuum concentrator (concentrator 5301, Eppendorf) until the solvent was completely evaporated. Afterwards, methanol (POCH) was added and methanol solutions were analyzed using gas chromatography and mass spectroscopy.

The GC/MS analyses were carried out on a Clarus 500 gas chromatograph (PerkinElmer)/Clarus 500 mass spectrometer (PerkinElmer). A 30 × 0.25 mm ID × 0.25 μm film Rtx®-200MS-fused silica capillary column (Restek) was used. The GC oven temperature was programmed as follows: initial temperature of 70 °C was held for 120 s, and then raised at 0.2 °C/s to 250 °C with a hold time of 120 s. The 1-mm^3^ injection volume was carried out in splitless mode at a temperature of 250 °C. The carrier gas was helium at a flow of 0.025 cm^3^/s. The mass spectrometer was operated in scan mode from 70 to 230 amu with a scan time of 40 ms, sir mode *m*/*z* = 194 amu of 100 ms and sir mode *m*/*z* = 197 amu of 100 ms.

## Results and conclusions

Table 3 contains the results of the determination of the following selected anthropogenic contaminations: caffeine concentration, nitrates, biochemical oxygen demand after 7 days, number of *E. coli*, number of *E. faecalis* in the water collected from different sampling spots in two sampling series.

**Table 3:** Caffeine concentration, physicochemical, and microbiological parameters in water samples from Rudawa Basin rivers

Sampling spot	26 June 2011			2 October 2011		
	Caffeine [ng/dm^3^]	Nitrate [mg/dm^3^]	BOD [mg/dm^3^]	Caffeine [ng/dm^3^]	*Escherichia coli* [CFU/100 cm^3^]	*Enterococcus faecalis* [CFU/100 cm^3^]
P1	380.1±34.3	14	1.7	113.0±6.3	>2420	227
P2	306.4±23.3	16	1.7	233.6±8.2	>2420	560
P3	163.2±13.3	11	1.8	108.3±20.7	649	186
P4	271.7±12.6	14	4.2	47.9±8.0	>2420	372
P5	67.5±6.0	19	1.3	14.4±4.8	488	108
P6	40.1±6.6	15	1.8	70.7±4.2	145	104
P7	116.1±18.7	14	1.6	46.9±1.7	>2420	1820
P8	114.2±1.6	17	1.7	27.1±4.3	579	152
P9	180.4±19.5	16	2.6	14.5±1.0	727	145
P10	273.6±6.3	16	2.8	45.6±6.2	>2420	198
P11	288.2±10.8	17	2.5	55.8±1.9	1986	168
P12	202.4±11.5	13	2.6	32.1±1.2	276	53

*CFU* colony forming units

Caffeine was detected in all samples investigated. In the first sampling series, the concentration ranged from 40.1 to 380 ng/dm^3^ and in the second sampling series, the concentration ranged from 14.4 to 233.6 ng/dm^3^. The values of the caffeine concentrations obtained during the second sampling series are lower than the results of the first series of measurements. Between the first and the second sampling series, there had been no rain for a long time and prolonged drought. In water samples taken during the first collection, the highest concentration of caffeine was determined in water from the Krzeszówka river (P1). At this point, Rudawa water contains water from three streams, the Eliaszówka, Czernka, and Miękinia. The lowest concentration of caffeine was in the water taken from the Będkówka (P6). In the second series of measurements, the highest concentration of caffeine was determined in the water obtained from the Filipówka stream (P2), and the lowest concentrations of caffeine were in water from the Rudawka stream (P5) and the Rudawa river (P9).

During the first series of measurements, two physical-chemical indicators of water quality were additionally investigated: the nitrate (nitrate (V)) concentration and biochemical oxygen demand (BOD). The nitrate concentrations varied from 11 to 19 mg/dm^3^ and the biochemical oxygen demand varied from 1.3 to 4.2 mg/dm^3^. The lowest value of the nitrate concentration was in the water from the Dulówka stream (P3), the highest in water from the Rudawka stream (P5). Water from the Rudawka (P5) had the lowest value of biochemical oxygen demand. The highest value of this indicator was in the water from the measuring point located in the lower Krzeszówka (P4). During the second series of measurements, determinations of microbiological parameters were made. In water from five sampling points (P1, P2, P4, P7, and P10), the number of *E. coli* was above 2420 colony forming units in 100 cm^3^. The lowest value of *E. coli* numbers were obtained for water from the Będkówka stream (P6). The number of *E. faecalis* was in the range from 53 to 1820 colony forming units in 100 cm^3^. The lowest value was obtained for water at the point near the Rudawa mouth on the Vistula (P12), the highest value was at the point located after the connection of the Bolechówka and Kobylanka streams (P7).

At 10 km of the Rudawa River, the water gauge station Balice is located. The caffeine flow rate at this point was estimated based on the value of the Rudawa River flow (www.krakow.pios.gov.pl/inform.php) and the concentration of caffeine in two sampling points which were near the Balice water gauge station (P10 and P11). The results of calculations of the daily mass flow rates of caffeine for these points are presented in Table 4.

**Table 4:** The daily mass flow rate of caffeine in Rudawa River

Sampling spot	26 June 2011		2 October 2011	
	Run-off [m^3^/s]	Caffeine amount [g/day]	Run-off [m^3^/s]	Caffeine amount [g/day]
P10	2.02	47.8	1.96	7.7
P11		50.3		9.4

## Concluding remarks

The obtained results showed that the river basin was polluted by anthropogenic wastes.

It has been shown that in recent years, water from the Rudawa is class A3 or does not meet the requirements of any class (in Poland, EU). The most frequently exceeded parameters are the total number of coliforms and the total number of fecal coliforms. The exceeded values of these two parameters prove that untreated wastewater is discharged to the water in the Rudawa River. These two parameters prove that sewage enters the water in the Rudawa River.

Waste generated in households which gets into soil and water through leaking septic tanks and waste disposal on fields is a source of contamination of surface waters. This situation is caused by a lack of sewage systems in the rural areas. There are new parts of the network, sewage treatment plants are being built and modernized but still (in 2014) there is a large disparity in the development of sewerage systems of particular Rudawa basin municipalities; moreover, in three municipalities less than 50 % of the population uses the sewerage system. Impurities flow in streams and rivers, kill the organisms that live in them, and are dangerous to humans.

The Rudawa river basin is composed of many small watercourses. Small streams have low flow of water especially during drought conditions and the risk of contamination of their water is high. At the same time, sewage outlets are discharged to some of the watercourses studied. In particular sewage treatment plants the singular contamination may be better disposed of than in others. All this may be the cause of the lack of correlations between tested indicators of water quality.

Caffeine concentrations in water samples taken in the same locations during the two samplings in 2011 were not strictly consistent. Results obtained in the second series of measurements were lower, and the distribution of concentrations changed due to changes in hydrological conditions during samplings. Prolonged drought before the second series of measurements reduced water quality, but at the same time a lack of rain caused smaller amounts of wastewater to reach the surface waters before the second samplings. This was seen for all the sampling location during all years of measurements.

The higher concentrations of caffeine in the summer than in the autumn time could be connected with the increased human activities in this locality during summer, mainly agriculture activity and recreation. Contamination of the river came from the land run-off and domestic wastewater disposal. The caffeine concentration was significantly higher in the most anthropogenically active area among the sampling locations study and in the points located closer to the agricultural field. The highest concentrations of caffeine were seen in the locations with the highest human activity and the densest populations of people. There is no correlation between the concentration of caffeine and hydrologic parameters such as surface water temperature, pH, or dissolved oxygen. They show the stability of the river water investigated (Jagoda et al. 2013).

The continuous presence of caffeine in Rudawa River water suggest the inflow of human domestic waste into this water. There is no allowable concentration of caffeine in surface water. There are no such limits for another pharmaceuticals and cosmetics despite the steadily increasing contamination of surface water by these substances.

Since the presence of caffeine in surface water is connected only with a human domestic waste, it is possible to combine chosen microbiological markers with the concentration of caffeine in surface water and have new anthropogenic marker of human domestic waste. We have decided to check if there is such possibility for caffeine and the value of concentration of nitrates (V), biochemical oxygen demand after 7 days, number of *E. coli* and number of *E. faecalis*, but our investigations have not showed any correlations between the concentrations of caffeine and the concentrations of other anthropogenic contaminants determined in water.

Further investigations for another markers, for instance nitrates (III), are necessary.
